# Degradable starch microspheres transarterial chemoembolization (DSM-TACE) in patients with unresectable hepatocellular carcinoma: results from the Prospective Multicenter Observational HepaStar Trial

**DOI:** 10.1007/s00330-024-11272-8

**Published:** 2024-12-19

**Authors:** Federico Collettini, Tomáš Andrašina, Peter Reimer, Wolfgang Schima, Christian Stroszczynski, Yasmina Lamprecht, Timo Alexander Auer, Tomáš Rohan, Moritz Wildgruber, Bernhard Gebauer, Max Masthoff

**Affiliations:** 1 Department of Radiology, Charité University Medicine Berlin Berlin, Charitéplatz 1, 10117 Berlin, Germany; 2 Berlin Institute of Health (BIH), Anna-Louisa-Karsch-Straße 2, 10178 Berlin, Germany; 3 Department of Radiology and Nuclear Medicine, University Hospital Brno and Masaryk University, Jihlavská 340/20, 625 00 Brno, Czech Republic; 4 Department of Radiology, Klinikum Karlsruhe, Moltkestraße 90, 76133 Karlsruhe, Germany; 5 Department of Diagnostic and Interventional Radiology, Göttlicher Heiland Krankenhaus, Dornbacher Straße 20-30, 1170 Wien, Austria; 6 Department of Radiology, University Medical Center Regensburg, Franz-Josef-Strauß-Allee 11, 93053 Regensburg, Germany; 7 Department of Radiology, University Hospital, LMU Munich, Marchioninistraße 15, 81377 Munich, Germany; 8 Clinic of Radiology, University Hospital of Münster, Albert-Schweitzer Campus 1, 48149 Münster, Germany

**Keywords:** Hepatocellular carcinoma, Liver cancer, Chemoembolization, Embolization

## Abstract

**Objectives:**

Despite increasing interest, prospective data on the use of degradable starch microsphere-transarterial chemoembolization (DSM-TACE) in the management of patients with unresectable HCC are still scarce. The objective of the HepaStar study was to collect prospective safety and effectiveness data in a prospective multicenter observational study.

**Materials and methods:**

Between January 2017 and December 2022, consecutive participants with unresectable or recurrent HCC treated with DSM-TACE as standard of care at 6 participating centers in Europe were enrolled. Tumor response was evaluated according to the mRECIST criteria. Overall survival (OS), progression-free survival (PFS), and adverse events (AEs) were assessed by using Kaplan–Meier analysis and Common Terminology Criteria for Adverse Events, version 5. Liver function deterioration was assessed by monitoring changes in liver blood tests during the follow-up.

**Results:**

Seventy-nine participants (median age, 69 years (IQR, 51–87 years); 67 men (85%)) were enrolled and treated. The median follow-up time was 18 months (IQR 9.5–38.0 months). The estimated median OS and PFS for the entire cohort was 32 months (CI, 95% 21-NaN) and 9 months (CI, 95% 7-NaN), respectively. Eleven (13.9%) participants experienced at least one grade 3 or 4 AE. The most frequent grade 3–4 AE was elevated bilirubin (2.2%, 5 of 79). Deterioration of bilirubin, AST, ALT, and albumin were observed in 24.1%, 23.7%, 19%, and 24% of participants, respectively.

**Conclusion:**

DSM-TACE achieves promising survival in patients with unresectable or recurrent HCC. This technique shows a favorable safety profile both in terms of treatment-related AEs and liver function deterioration.

**Key Points:**

***Question*** *Although degradable starch microspheres transarterial chemoembolization is widely used in clinical practice across Europe, prospective data on its application in hepatocellular carcinoma patients remains limited.*

***Findings*** *Degradable starch microspheres transarterial chemoembolization results in promising survival rates, good tumor response rates, and low rates of treatment-related adverse events.*

***Clinical relevance*** *In patients with unresectable hepatocellular carcinoma, degradable starch microspheres transarterial chemoembolization represents a safe and effective alternative to more well-established chemoembolization techniques like conventional transarterial chemoembolization and drug-eluting beads transarterial chemoembolization.*

## Introduction

Hepatocellular carcinoma (HCC) is the world’s third leading cause of cancer-related death, with increasing incidence rates and cancer-specific mortality in many countries [1]. Unfortunately, most patients are diagnosed at stages, where ablation, resection, and transplantation are no longer possible curative treatment options [2]. For such patients, transarterial therapies represent an option, with transarterial chemoembolization (TACE) being advocated as the primary therapy for individuals with intermediate-stage (Barcelona Clinic Liver Cancer (BCLC) stage B) HCC according to the European Association for the Study of the Liver guidelines [3]. Currently, the most frequently employed methods are conventional transarterial chemoembolization (cTACE) and drug-eluting beads transarterial chemoembolization (DEB-TACE) [4, 5].

Although accessible for many years, TACE utilizing degradable starch microspheres (DSM) as embolic agents has only recently gained more widespread clinical adoption in Europe. DSMs are composed of polymerized, hydrolyzed starch molecules linked by glycerol ether groups and are readily broken down by blood α-amylase, with a half-life of approximately 35–50 min both in vitro and in vivo [6]. As a result, DSM-TACE is characterized by a well-defined and, most importantly, transient, relatively short-lasting vessel occlusion [7]. Such temporary embolization offers numerous potential advantages in the context of TACE: First, it facilitates the option for further treatment, as the vessels’ early reopening enables timely transarterial re-interventions [8]. Second, reducing ischemic time minimizes damage to healthy liver tissue in case the tumor cannot be reached super selectively, preventing deterioration of liver function [7, 9–11]. Hence, as unselective, lobar non-permanent embolization can be performed with high tolerability and safety rates, DSM-TACE represents a viable option for patients with extensive or bilobar disease or situations, where selective treatment is not feasible [12, 13]. Accordingly, the current standard of practice guidelines for the treatment of HCC by the Cardiovascular and Interventional Radiological Society of Europe (CIRSE) mention DSM-TACE among available TACE techniques [14]. DSM-TACE is portrayed as particularly suitable for the treatment of patients with multifocal tumors, more advanced disease states (including patients with portal vein thrombosis), and patients with borderline preserved liver function (bilirubin >  3 mg/dL) [14]. Despite increasing interest in DSM-TACE for the treatment of HCC, prospective data regarding the use, safety, and effectiveness of this technique are still scarce. The objective of the HepaStar study was to collect prospective data on the mode of application, safety, and effectiveness of DSM-TACE in HCC treatment.

## Materials and methods

### Study design and participants

Between January 2017 and December 2022, consecutive participants with unresectable HCC treated with DSM-TACE as standard of care at one of the six participating centers in three European countries were enrolled in the prospective, multicenter, observational HepaStar study. The study protocol did not prescribe or encourage the use of DSM-TACE in a particular patient group but observed its use in the real-life clinical setting. The study was approved by the institutional review board (EA1/359/16). All participants signed informed written consent. The study was performed in accordance with the ethical standards of the institutional research committee and with the 1964 Helsinki Declaration and its later amendments. Key inclusion criteria were: (1) HCC diagnosed by biopsy or according to the American Association for the Study of Liver Disease (AASLD) criteria for non-invasive HCC diagnosis, (2) tumor unsuitable for curative treatments or where curative treatments have failed (progressive or recurrent HCC after resection or ablation). Key exclusion criteria included: (1) Previous or present systemic HCC treatment, (2) history of transarterial chemoembolization (TACE) at any point in the past, (3) history of liver radiation therapy including radiolabeled microspheres (Y-90 Radioembolization) at any point in the past, (4) DSM-TACE performed as part of a combined treatment with ablation, and (5) presence of brain metastases.

### DSM-TACE procedures

Under local anesthesia, the right or left common femoral artery was punctured in the Seldinger technique, and a 4 or 5F angiographic sheath was placed to secure the vascular access site. Selective angiograms of the coeliac axis and the superior mesenteric artery were performed to assess the tumor-supplying arteries. In selected cases and based on the treating center’s preferences, an additional contrast-enhanced cone-beam CT (CBCT) was conducted to identify the tumor-feeding artery. Subsequently, a coaxial microcatheter system was advanced in the tumor-supplying branches of the hepatic artery. As DSM-TACE can be performed both super selectively and non-selectively depending on the number and localization of the tumors to be treated, centers were asked to document the application site. Even for lobar administration, the use of a microcatheter was preferred to reduce the risk of vasospasm and arterial dissection. Once correct positioning of the microcatheter was confirmed, a suspension of 450 mg/7.5 mL of DSM (EmboCept^®^ S, PharmaCept/EmboCept^®^ S DSM 50 µm, Magle PharmaCept) was mixed with the chemotherapeutic drug (e.g., 50 mg/25 mL of doxorubicin) and 17.5 mL of contrast agent, as recommended by the manufacturer. The suspension was slowly and continuously administered under fluoroscopic control. When DSM-TACE was performed in a lobar fashion, the endpoint of embolization was a “tree-in-the-winter” appearance with occlusion of small tumor-feeding vessels but preservation of flow in the major lobar and segmental arteries. For cases in which more than 450 mg of DSM was needed to achieve sub-stasis, a second vial of DSM was used until a maximum dose of 900 mg.

### Follow-up and efficacy evaluation

At the time of first treatment, baseline demographic data and treatment-related data were collected. All participants were tracked until death, last follow-up, or the conclusion of the study.

Specific follow-up intervals were left to the discretion of the treating center. It was recommended that patient follow-up data, including tumor response at imaging and toxicity, were collected at 1, 3-, and 6-month follow-up visits. Tumor response was evaluated by multidetector computed tomography or magnetic resonance imaging (MRI), according to the modified RECIST (mRECIST). Objective response rate (ORR) was defined as the sum of complete and partial responses. The disease control rate (DCR) was defined as the sum of the objective response rate plus stable disease. Progression-free survival (PFS) was defined as the time from the first DSM-TACE until tumor progression at imaging or death. Overall survival (OS) was measured from the day of the first DSM-TACE until the date of death for any cause. PFS and OS were calculated by censoring participants who underwent orthotopic liver transplantation during follow-up. Survival status was collected as information became available or at the end of the study.

### Adverse events and liver deterioration

Adverse events (AEs) were monitored and recorded in dedicated case report forms during and after each treatment, and at all follow-up visits and graded according to the Common Terminology Criteria for Adverse Events, version 5. Furthermore, liver function deterioration was also assessed by monitoring changes in baseline serum bilirubin, serum albumin, aspartate transaminase (AST), and alanine transaminase (ALT) values during the follow-up. The laboratory value prior to the first DSM-TACE was used as the baseline value. To estimate liver function deterioration, the last available laboratory value was used (30–180 days after the first TACE). Due to the lack of universally accepted liver function deterioration criteria, clinically meaningful changes in laboratory parameters were assessed according to the definition adopted by Miksad et al in the LiverT study (Liver function thresholds to establish clinically meaningful deterioration after DSM-TACE are reported in Table S1 in the electronic supplementary material [15].

### Statistical analysis

Data are presented as mean ± standard deviation or median (interquartile range (IQR)) for continuous variables and numbers (%) for categorical variables. Percentages are based on the whole cohort unless otherwise indicated. Participants who died during the study were defined to have progression regarding PFS. Participants alive and event-free were censored on the day of the last follow-up. Kaplan–Meier curves with 95% CIs were constructed for PFS and OS. Laboratory results were described in absolute values (mean and standard deviation, and median with IQR). A two-sided pairwise Wilcoxon sign-rank test was used to generate *p*-values testing the null hypothesis that the median difference in laboratory values between two-time points is zero. The incidence of deterioration for each laboratory value from baseline (prior to the first DSM-TACE) to the chronic period (30–180 days after the first TACE) was calculated based on the total population and reported with a 95% confidence interval (CI). For all calculations, *p* < 0.05 was considered indicative of a statistically significant difference. Calculations were performed by T.A.A. using R software, version 4.1 (R Foundation for Statistical Computing).

## Results

### Study cohort

Seventy-nine participants with unresectable HCC from six centers in three European countries were included in this study. Participant baseline demographics are outlined in Table 1. Briefly, the mean age was 69 years, and 67/79 (85%) participants were male. Most of the participants had intermediate-stage HCC (55/79, 70%), Eastern Cooperative Oncology Group (ECOG) performance status 0 (52/79, 66%), and well-compensated liver disease (Child-Pugh A in 59/79, 75%). Extra-hepatic disease was diagnosed in 3/79 (4%) participants, and portal vein tumor thrombosis was present in 5/79 (6%). Most participants had bi- or multifocal disease (52/79, 66%) with a mean nodule count of 3.2 (range: 1–15). The mean diameter of the largest HCC lesion was 4.4 cm (range: 1–14 cm). Fifteen of 79 participants (19%) had an HCC relapse after a previous curative treatment. Seven of 79 (9%) participants had an intrahepatic relapse following hepatic resection, 6/79 (8%) participants had a relapse after previous ablation, and 2/79 (2.5%) had previously undergone both hepatic resection and ablation.

**Table 1:** Baseline demographic and clinical characteristics of participants

Category	Subcategory	*n* = 79
Sex	Male	67 (85%)
	Female	12 (15%)
Age (years)	Mean ± SD	69 ± 8.34
	Median (IQR)	69 (51–87)
ECOG performance status	0	52 (66%)
	1	26 (33%)
	2	1 (1%)
Cirrhosis	Yes	73 (92%)
	No	6 (8%)
Cause of cirrhosis	Alcohol use disorder	43 (59%)
	Viral	16 (21.9%)
	NASH	4 (5.4%)
	Biliary disease	1 (1.4%)
	Cryptogenic	9 (12.3%)
BCLC Stage	A	16 (20%)
	B	55 (70%)
	C	8 (10%)
Child-Pugh score	A	59 (75%)
	B	20 (25%)
Portal vein tumor thrombosis	Yes	5 (6%)
	No	74 (94%)
Previous curative treatments	None	64 (81%)
	Hepatic resection	7 (9%)
	Ablation	6 (8%)
	Hepatic resection & ablation	2 (2%)
Tumor burden	Unilobar	48 (61%)
	Bilobar	31 (39%)
Tumor load	Solitary	27 (35%)
	Bifocal	20 (25%)
	Multifocal	32 (40%)
Nodule count	Mean ± SD	3.2 ± 3,0
	Median (IQR)	2.0 (1–4)
Largest nodule size (cm)	Mean ± SD	4.4 ± 2.8
	Median (IQR)	3.6 (2.5–5.6)

### Procedures

In total, 242 DSM procedures were performed during the observation period. The observation time ended on 31 December 2023. Participants underwent a mean of 3 (range: 1–12) DSM-TACE procedures (Table 2). DSM-TACE procedures were administered at intervals of approximately 6 weeks (median interval: 43 days). Lobar treatment was the most common treatment (160/242, 66%), followed by segmental/super selective treatment (75/242, 31%). Seven of 242 procedures (3%) were performed as whole-liver treatment with the tip of the catheter in the proper hepatic artery. 241 of 242 (99.6%) were performed using a coaxial technique with a microcatheter. Intraprocedural cone-beam computed tomography (CBCT) was performed in 124/242 (51%) of procedures. Doxorubicin was used as a chemotherapeutic agent in 240/242 (99%) of the procedure; the remaining 2/242 (1%) procedures were performed with epirubicin. The mean doxorubicin dose applied was 47.5 mg (range: 29–50 mg); the mean epirubicin dose was 75 mg (range: 50–100 mg). All DSM-TACE procedures were performed with DSM as the only embolic material. The mean DSM dose was 425.8 mg (range: 150–900 mg). Follow-up imaging was performed with contrast-enhanced CT in 53% (102/191) of cases and with contrast-enhanced MRI in 46% (89/191) of cases.

**Table 2:** Treatment parameters

Parameter	Subcategory	*n* = 242
Embolization technique	Monoaxial	1 (0.4%)
	Coaxial	241 (99.6%)
Site of embolization	Proper hepatic artery	7 (3%)
	Lobar	160 (66%)
	Super selective	75 (31%)
Intraprocedural CBCT	Yes	124 (51%)
	No	118 (49%)
Chemotherapy	Doxorubicin	240 (99%)
	Epirubicin	2 (1%)
Doxorubicin dosage	Mean (range)	47.5 mg (29–50 mg)
Epirubicin dosage	Mean (range)	75 mg (0–100 mg)
DSM dosage	Mean (range)	425.8 mg (150–900 mg)
Follow-up imaging technique	Computed tomography (CT)	102/191 (53%)
	Magnetic resonance imaging (MRI)	89/191 (46%)

### Adverse events

No treatment-related death was observed. Table 3 shows treatment-related AEs and their corresponding Grades. Eleven of 79 (13.9%) participants experienced at least one grade 3 or 4 AE. The most frequent grade 3–4 AEs were biologic: elevated bilirubin (2.2%, 5 of 79) and elevated g-glutamyl transpeptidase (0.8%, 2 of 79). Grade 3 pain, elevated AST, and elevated ALT were documented in 1 participant (0.4%), respectively. One participant (0.4%) experienced Grade 4 tumor bleeding that was successfully treated with transarterial embolization. No cases of DSM-TACE discontinuation due to toxicity were recorded.

**Table 3:** Treatment-related AEs

Adverse events	All grades	Grades 1–2	Grade 3	Grade 4
Elevated bilirubin	55 (24%)	50 (21.8)	5 (2.2)	0 (0.0)
Vomiting/nausea	8 (3.5%)	8 (3.5)	0 (0.0)	0 (0.0)
Pain	8 (3.5%)	7 (3.1)	1 (0.4)	0 (0.0)
Elevated GGT	8 (3.5%)	6 (2.6)	2 (0.8)	0 (0.0)
Degraded albumin	7 (3%)	7 (3.1)	0 (0.0)	0 (0.0)
Elevated AST	6 (2.6%)	5 (2.2)	1 (0.4)	0 (0.0)
Hypertension	4 (1.7%)	4 (1.7)	0 (0.0)	0 (0.0)
Elevated ALT	2 (0.8%)	1 (0.4)	1 (0.4)	0 (0.0)
Bleeding	1 (0.4%)	0 (0.0)	0 (0.0)	1 (0.4)

### Liver function following DSM-TACE

**Fig. 1:**
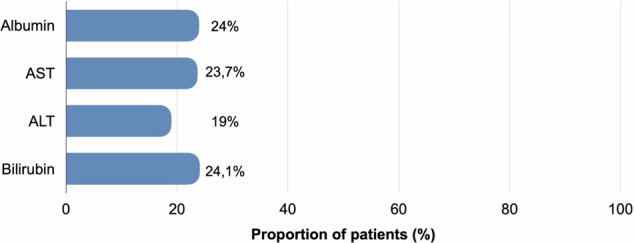
Proportion of participants with chronic liver function deterioration after DSM-TACE compared to baseline. Deterioration thresholds: bilirubin increase of ≥ 50%, albumin decrease by 0.3 g/dL, AST increase of > 25%, ALT increase of > 25%, all compared with baseline. ALT, alanine transaminase; AST, aspartate transaminase; CI confidence interval

During the first 6 months of observation, participants underwent a mean of 2.8 DSM-TACE procedures (range: 1–6). At the last available follow-up, 30–180 days after the first DSM-TACE, a statistically significant impact of DSM-TACE on median laboratory values was evident only for bilirubin (Table 4). Deterioration of bilirubin, AST, ALT, and albumin were observed in 24.1%, 23.7%, 19%, and 24% of participants, respectively (Fig. 1).

**Table 4:** Liver function tests

Laboratory parameter	Baseline value	Last value	*p*-value
Bilirubin			
Median (IQR)	0.7 (0.54–1.11)	0.98 (0.60–1.56)	0.002
Serum albumin			
Median (IQR)	39 (34.8–43)	38.3 (33.9–41)	0.349
AST			
Median (IQR)	50 (35.6–66.8)	48 (34–76)	0.899
ALT			
Median (IQR)	36 (23–51)	31.8 (22–47.5)	0.625

### Follow-up and oncologic outcomes

**Fig. 2:**
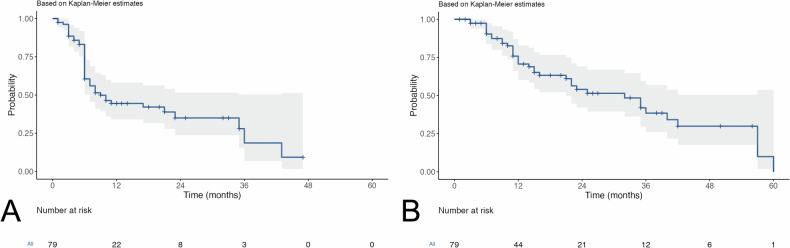
Kaplan–Meier plots show entire cohort (**A**) progression-free survival (PFS), and (**B**) overall survival (OS)

The median follow-up time was 18 months (IQR 9.5–38.0 months). Response data was available in 63/79 participants (79,7%) at a 1-month visit, in 70/79 (88.6%) at a 3-month visit, and in 58/79 (73.4%) participants at a 6-month visit (Table 5). The ORR was 27% at 1 month, 45% at 3 months, and 36% at 6 months. The corresponding DCR was 97% at 1 month, 94% at 3 months, and 67% at 6 months. 13/79 (16.5%) participants underwent liver transplantation during follow-up. The mean and median time to liver transplantation were 8.7 ( ± 5.8) and 6.0 (IQR, 5.0–11.0) months, respectively. The median estimated PFS with censoring for orthotopic liver transplant was 9 months (CI, 95% 7-NaN) (Fig. 2A). Estimated 1-year, 2-year, and 3-year PFS rates were 44.5% (CI, 95% 34–58.2), 35% (CI, 95% 24–51.6), and 18.7% (CI, 95% 7–50.3). During the observation period, 40/79 (50%) participants died, and 39/79 (50%) were still alive. Estimated median, 1-year, 2-year, and 3-year OS with censoring for orthotopic liver transplant were 32 months (CI, 95% 21-NaN), 70.7% (CI, 95% 60.3–82.8), 54.0% (CI, 95% 42.2–69.2), and 38.5% (CI, 95% 26.0–56.9), respectively (Fig. 2B).

**Table 5:** Tumor response at imaging

Follow-up visit	Complete response	Partial response	Stable disease	Progressive disease
1-mo response (*n* = 63)	6 (10%)	11 (17%)	44 (70%)	2 (3%)
1-mo ORR	17 (27%)			
1-mo DCR	61 (97%)			
3-mo response (*n* = 70)	10 (14%)	22 (31%)	34 (49%)	4 (6%)
3-mo ORR	32 (45%)			
3-mo DCR	66 (94%)			
6-mo response (*n* = 58)	9 (16%)	12 (20%)	18 (31%)	19 (33%)
6-mo ORR	21(36%)			
6-mo DCR	39 (67%)

## Discussion

The HepaStar study was designed to assess safety and effectiveness outcomes after DSM-TACE in a real-life cohort of HCC participants across different European centers. To the best of our knowledge, this study is the first prospective multicenter cohort on the use of DSM-TACE for the treatment of unresectable HCC with structured reporting of oncologic outcome and toxicity data.

One of the main purposes of the study was to elucidate the safety profile of DSM-TACE in a real-life cohort of participants with unresectable HCC. Our results indicate that DSM-TACE has a favorable safety profile with minimal impact on liver function, which may be beneficial, especially in patients with bilobar and multifocal disease. In fact, no treatment-related death and only one (0.4%) grade 4 AE was observed. Here, a patient with an exophytic HCC experienced tumor bleeding one day after DSM-TACE and was successfully treated with transarterial embolization. 11.4% of participants experienced at least one grade 3 AE, with bilirubin elevation being the most frequent grade 3 AE (2.2%, 5 of 79). The data on liver function deterioration is particularly revealing. Despite participants undergoing an average of 2.8 DSM-TACE procedures within the first 6 months of observation and most procedures being performed in a lobar manner, less than 25% of participants exhibited significant deterioration in liver function tests indicative of a reduction of hepatic reserve. These findings are promising, especially when compared to the LiverT study, where similar levels of deterioration were observed after only a single TACE procedure [15].

Regarding tumor response according to mRECIST, we observed an ORR of 27% at 1 months, 45% at 3 months and 36% at 6 months while the corresponding DCR was 97%, 94% and 67%, respectively. These results are in line with the results of previous studies reporting similar ORR and DCR in HCC patients treated with DSM-TACE. In a study on 50 patients, Schicho et al reported an ORR of 44% and DCR of 70% at 1 month [16]. In a smaller study on 28 HCC patients treated with at least 3 DSM-TACE procedures, Haubold et al reported an ORR of 39.3% and a DCR of 78.6% at 3 months, while Niessen et al reported an ORR of 44.1% and DCR of 82.4% at 6 months in 34 patients [17, 18]. However, our data shows a lower ORR at 1 month compared to a prospective single-center study by Orlacchio et al (ORR of 84.3%), while DCR (98.9%) was similar to our data [10]. Reported ORR after cTACE and DEB-TACE are higher. The PRECISION V study, a multicenter, prospective, randomized phase II study including 212 patients comparing DEB-TACE and cTACE, reported a 6-month ORR of 51.6% or 43.5% and a DCR of 63.4% or 51.9% [19]. Similarly, the PRECISION Italia, a multicenter, prospective, randomized active-controlled study including 177 patients, found a higher ORR at 1-, 3-, and 6-months for both DEB-TACE (89.9%, 74.7%, and 59.7%) and cTACE (88.5%, 74.1% and 70.3%), while DCR were similar to lower at 1-, 3-, and 6-months for DEB-TACE (93.3%, 79.7%, 65.7%) and cTACE (92.0%, 77.8%, 73.0%) compared to our data on DSM-TACE. The differences in tumor response seem to be more pronounced in the short-term follow-up and are likely due to the different embolization techniques. In fact, while cTACE and DEB-TACE are routinely conducted susperselectively, in our study, 61% were performed using a lobar or whole-liver approach, which is known to impact OS after TACE.

The estimated median OS with censoring for orthotopic liver transplant was 32 months (CI, 95% 21-NaN) with estimated 1-year, 2-year, and 3-year OS rates of 70.7% (CI, 95% 60.3–82.8), 54.0% (CI, 95% 42.2–69.2), and 38.5% (CI, 95% 26.0–56.9), respectively. The survival data reported in this study are quite promising and compare favorably with reported median OS figures after cTACE and DEB-TACE in real-life cohorts. In a large systematic review of trials published between January 1, 1980, and June 30, 2013, and including a total of 10,108 patients treated with lipiodol TACE, Lencioni et al reported a median OS of 19.4 months (95% CI: 16.2–22.6) and OS rates of 70.3% at 1 year, 51.8% at 2 years and 40.4% at 3 years. In a more recent, multicentric studies, Han et al and Auer et al reported median OS of 19.9 months and 21.8 months, respectively, almost identical to the figures reported by Lencioni et al [20–22]. Further, the PRECISION Italia trial reported an estimated median OS of 28 months for cTACE patients and 29 months for DEB-TACE patients [23]. A possible explanation for these favorable survival rates when using DSM for TACE may be attributed to the advantageous safety profile of DSM. Although there are no comparative studies describing the lower toxicity of DSM-TACE compared with other techniques such as cTACE or DEB-TACE, we hypothesize that the short-term transient vessel occlusion may minimize the damage to non-tumor liver tissue preventing deterioration of liver function, especially in cases where the tumor cannot be reached super selectively.

Another important goal of the HepaStar trial was to work out the role of DSM-TACE in the clinical practice of European centers that experienced this type of treatment. The baseline characteristics of the participants included in the study demonstrate that although DSM-TACE is generally portrayed as particularly suitable for the treatment of patients with multifocal tumors, more advanced disease states (including patients with portal vein thrombosis) and patients with borderline preserved liver function (bilirubin > 3 mg/dL), experienced centers tend to use this technique across the spectrum of unresectable HCC by performing super selective treatments in cases of circumscribed tumors and lobar treatments or even whole-liver treatments in patients with bilobar and multifocal tumors. These important aspects demonstrate the flexibility and versatility of this technique, which have the potential to substantially reinforce the use of TACE across the broad spectrum of patients with intermediate-stage disease.

Several limitations of the HepaStar trial need to be acknowledged. First, despite the multicenter design, the study cohort size is still limited, and the included patient populations are heterogeneous. The heterogeneity of the population, although inherent in the very nature of an observational study without strict inclusion criteria, while giving us a good indication of the capability of this technique in a real-life scenario, limits the conclusions that can be drawn for patients with specific characteristics. Secondly, also because of the COVID-19 pandemic that occurred during the study period the lost to follow-up rate in this study was around 10 to 25% depending on the time of follow-up visit, resulting in a potential bias of reported data. Thirdly, the study was not designed as a comparative study of DSM-TACE versus DEB- and/or cTACE and thus does not aim to compare these techniques. How DSM-TACE compares to other TACE techniques remains an open question, which needs to be addressed in future comparative studies. Instead, this study was designed to analyze the safety, effectiveness, and survival after DSM-TACE in HCC in a prospective and multicenter manner, considering the current lack of such data for this technique.

In conclusion, the results of the HepaStar trial show that DSM-TACE is a highly versatile technique with a favorable safety profile both in terms of treatment-related AEs and in terms of liver function preservation. DSM-TACE achieves good response rates and survival rates in real-world patients with unresectable HCC. Moreover, we believe that the distinctive characteristics of this technique make it particularly suitable for incorporation into combination regimens with systemic immunotherapies. The short ischemic time, combined with the versatility and tolerability of DSM-TACE, positions it as a promising component in such therapeutic approaches. Therefore, pursuing prospective studies aimed at evaluating the efficacy and safety of this technique within combination regimens is strongly recommended.

## Supplementary information

ELECTRONIC SUPPLEMENTARY MATERIAL

## Abbreviations

AE: Adverse events
ALT: Alanine transaminase
AST: Aspartate transaminase
CBCT: Cone beam computed tomography
CI: Confidence interval
DCR: Disease control rate
DEB: Drug-eluting beads
DSM: Degradable starch microspheres
ECOG: Eastern Cooperative Oncology Group
HCC: Hepatocellular carcinoma
IQR: Interquartile range
mRECIST: Modified Response Evaluation Criteria in Solid Tumors
MRI: Magnetic resonance imaging
ORR: Objective response rate
OS: Overall survival
PFS: Progression-free survival
TACE: Transarterial chemoembolization
