# Supplementary material for: Degradable starch microspheres transarterial chemoembolization (DSM-TACE) in patients with unresectable hepatocellular carcinoma: results from the Prospective Multicenter Observational HepaStar Trial
Source: Eur Radiol. 2024 Dec 19;35(7):4132–40. doi: 10.1007/s00330-024-11272-8 (PMC12165977; doi:10.1007/s00330-024-11272-8)
Supplement: Supplementary file 1 — ELECTRONIC SUPPLEMENTARY MATERIAL [file 330_2024_11272_MOESM1_ESM.pdf]

Degradable Starch Microspheres Transarterial Chemoembolization (DSM-TACE) in patients with unresectable Hepatocellular Carcinoma: Results from the Prospective Multicenter Observational HepaStar Trial.
ELECTRONIC SUPPLEMENTARY MATERIAL

Table 1 Liver function thresholds

Liver function thresholds to establish clinically meaningful deterioration after DSM-TACE.

Parameter	Deterioration threshold (change from baseline)
Serum total bilirubin	Increase of ≥ 50%
Serum albumin	Decrease by ≥ 0.3 g/dL
AST	Increase of > 25%
ALT	Increase of > 25%
